# ARIMA based projection of infant mortality rate by the year 2030: a comparative analysis of India and Madhya Pradesh

**DOI:** 10.1186/s12889-024-21073-9

**Published:** 2025-08-07

**Authors:** Abhinav Bahuguna, Akanksha Uniyal, Vidisha Vallabh

**Affiliations:** 1 Department of Biostatistics, Himalayan Institute of Medical Sciences, Swami Rama Himalayan University, Dehradun, Uttarakhand India; 2 Department of Community Medicine, Himalayan Institute of Medical Sciences, Swami Rama Himalayan University, Dehradun, India

**Keywords:** Infant mortality rate, Autoregressive integrated moving average, Akaike’s information criterion, Bayesian information criterion, Decadal growth rate, Mean absolute percentage error, India, Madhya Pradesh

## Abstract

**Background:**

Infant mortality is an important predictor of a government’s commitment to its people. Global infant deaths have declined since past decades but at a pace that leaves much to be desired. India’s declining pattern of trends is encouraging but the low performance of individual states like Madhya Pradesh (MP) indicates an urgent need for policy revision and implementation.

**Methods:**

This paper forecasts the Infant mortality rate (IMR) of India and MP by the year 2030 through autoregressive integrated moving average (ARIMA) model after obtaining stationarity by differencing the series of IMR once. The Akaike’s information criterion and Bayesian information criterion have been used for the selection of best ARIMA model amongst other existing choices. The model diagnostics through Ljung and Box test shows absence of autocorrelation in the residuals (*p* > 0.05).

**Results:**

The findings through ARIMA(3, 1, 0) foretell a declining IMR from 27 to 20 per thousand live births (from 2021 to 2030) in India. Similarly, MP is expected to experience reduction in infant deaths from 44 to 39 per thousand live births (from 2021 to 2030). The deployed model is well fitted as mean absolute percentage error lies below 5%. During 2010–20, India and MP witnessed a decadal reduction of 40% and 31% in IMR, respectively. From the year 2010 onwards, India experienced the highest annual reduction of 8.1% in IMR during 2015–16. Similarly, MP encountered a decrease of 6.5% in IMR recently (2019–20), which is the highest declining annual IMR in the state during past ten years.

**Conclusions:**

The projected figures on IMR are satisfactory for policy makers at national level, but MP is still miles away to achieve acceptable IMR as compared to the country’s IMR. The state requires more attention and focus on exploring reasons and identifying underlying factors responsible for higher IMR across its demographic structure including socio-economic characteristics.

## Introduction

Defined as “the number of infant deaths per 1000 live births”, the infant mortality rate (IMR) is a significant indicator of comprehensive health of children and a government’s vested interest in its vulnerable population [1]. Globally, pneumonia, diarrhoea, birth defects and malaria majorly contribute to death of children under 5 years of age. Most deaths in children occur in the first year of birth, more so in the first month where premature births, maternal pregnancy complications (asphyxia/trauma), low birth weight, and acute respiratory infections are the main causes [2]. Determinants like socioeconomic development, basic living conditions, and environmental conditions deeply entwined with deaths in children. For this reason, mortality rates especially among children have been incorporated while devising developmental goals for countries all over the world [3].

The advent and progress of the Millennium Development Goals (MDGs) were reflected in the decline in under-five deaths globally. Despite a 4% decline in under-five mortality rate (U5MR) in the ultimate years globally, we failed to achieve MDG-4 i.e., U5MR of less than 30 deaths per 1000 live births [4]. The global efforts towards a two-thirds reduction in under-five deaths, neonate deaths were impeded by a slower decline in neonatal mortality rates (NMRs) especially early neonatal mortality rates (ENMRs) [5–7]. Since neonatal mortality contributes to majority of infant mortality, a decline in associated IMRs is seen (from 65 infant deaths per 1000 live births (1990) to 29 infant deaths per 1000 live births (2018)); it varies widely from country to country [8, 9].

Succeeding MDGs, the Sustainable Development Goals (SGDs) have aimed to reduce U5MRs and NMRs worldwide, leading to a reduction in IMR [10]. India is currently on target for reaching the SDG Goal 3.2 for newborn and childhood mortality because of the multitude of health programs focusing on childhood mortality [11, 12]. Facility-based Newborn Care, Mother’s Absolute Affection (MAA), Social Awareness and Actions to Neutralize Pneumonia Successfully (SAANS) are among the newer initiatives to decrease infant mortality [13].

The reduction in child mortality in India varies drastically despite initiatives being taken all over the country; the difference is based on state, districts, religion, caste, gender, place of living (rural or urban), and other determinants [7, 14]. Many states in India have attained the SDG targets for NMR (less than 12/ 1000 live births) viz. Kerala (4/1000 live births), Delhi (9/1000 live births), Tamil Nadu (9/1000 live births), Maharashtra (11/1000 live births), Jammu & Kashmir (12/1000 live births) and Punjab (12/1000 live births); and U5MR (less than 25/ 1000 live births) viz. Kerala (8/1000 live births), Tamil Nadu (13/1000 live births), Delhi (14/1000 live births), Maharashtra (18/1000 live births), Jammu and Kashmir (17/1000 live births), Karnataka (21/1000 live births), Punjab (22/1000 live births), West Bengal (22/1000 live births), Telangana (23/1000 live births), Gujarat (24/1000 live births), and Himachal Pradesh (24/1000 live births) [15]. States of MP, Uttar Pradesh (UP), Uttarakhand, Jharkhand, Orissa, Rajasthan, Bihar, Chhattisgarh and Assam constitute the low performance states with regards to NMR and U5MR goals for SDGs [16, 17]. In fact; UP, MP, Bihar and Rajasthan constitute about 55% and 15% of neonaal deaths across India and worldwide [7]. The dismaying performance of MP is comparable to poorer nations of the world [18–20]. The state presents a dismal picture on NITI Aayog’s Health Index [21].

To reach the goals together as one nation, it becomes imperative to chart future predictions regarding infant mortality by creating statistical analytical models. The exponential smoothing, autoregressive integrated moving average (ARIMA), seasonal autoregressive integrated moving average (SARIMA), vector autoregressive (VAR), vector error correction (VEC), and prophet model, etc., are examples of such time series models. The aim of the present paper is to project IMR of India and MP by the year 2030 using ARIMA model. The choice of ARIMA among above mentioned models lies in the fact that it is one of the most popularly used predictive model among epidemiologists because of its wide applicability and ease of execution. For a comparative study at national level, we have considered MP for our study because it is the lowest performing state in terms of IMR reduction over the years. Since prediction of any demographic event like IMR can be affected by several factors including population fluctuations, and socio-economic factors, therefore, selection of predictive model which can predict outcome variable accurately becomes important. This study utilises ARIMA process, which is based on assumption of stationarity of series having a large number of past observations to predict future values of IMR along with 95% prediction interval (PI) [22]. To select best ARIMA model, two well recognised statistics; say Akaike’s information criterion (AIC) and Bayesian information criterion (BIC) have been used. The lower numerical values of these yield to best outperforming model, among others. The prediction of IMR, is helpful as this can serve as a guide to policy makers for evaluation and tailoring of national health programs and policies as per the need of the nation and a particular state. As a contributor of one-fifth of global newborn deaths world-wide, its high time that country focusses not only on reduction of child mortality but also on sustainable methods of achieving the same [23, 24].

## Materials and methods

### Dataset and study area

**Fig. 1:**
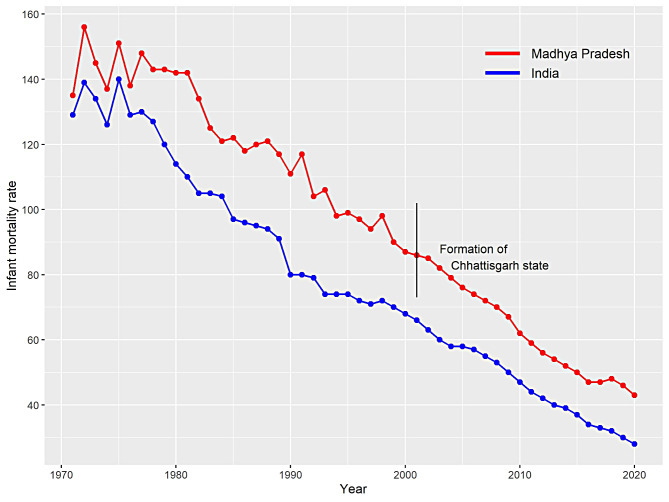
Declining trend of IMR of India and Madhya Pradesh from 1971 to 2020

The study utilises time series data on IMR of India and MP from 1971 to 2020, i.e., over the period of 50 years. The dataset is publicly available at “open government data (OGD) platform India” website [25]. Also, one can find the statistical report on the annual estimates of IMR in sample registration system (SRS), released by the Registrar General and Census Commissioner of India [26]. The study area for this research work includes India and MP, a state sharing highest percentage of infant deaths to overall country’s IMR during past years. A comparative study is made between India and MP. Figure 1 illustrates that situation has improved for India and MP over time with a sustained decreasing trend in IMR. However, it is important to note that the OGD platform discusses the real data on IMR but forgoes changes in population dynamics of MP after the formation of Chhattisgarh state from it in the year 2000.

### Statistical analysis

We have used R software version 4.2.3 for data analysis purpose. A time series model, proposed by Box and Jenkins (1970), ARIMA, is used to forecast IMR of India and MP based on available historical data. The mathematical expression for ARIMA model is given by;

1 $$\eqalign{& {y_t} = {\theta _0} + {\phi _1}{y_{t - 1}} \ldots \ldots \ldots + {\phi _p}{y_{t - p}} \cr & + {\theta _1}{{\text{\euro }}_{t - 1}} + \ldots \ldots + {\theta _q}{{\text{\euro }}_{t - q}} + {{\text{\euro }}_t} \cr}$$

where, $\:{\theta\:}_{0}$ denotes the intercept term, ${\phi _i}{\text{'s}}$ and ${\theta _j}{\text{'s}}$ are coefficients of autoregressive (AR) and moving average (MA) terms respectively. The model defined in expression (1) is expressed as ARIMA(p, d, q), where ‘p’ and ‘q’ are order of AR and MA terms of any ‘d^th^’ times differenced series corresponding to an original time series of observations. ${\text{\euro }}$ is identically independently distributed error term with ‘zero’ mean and ‘constant’ variance [27].

The Augmented Dickey-Fuller (ADF) and Kwiatkowski-Phillips-Schmidt-Shin (KPSS) tests have been applied on IMR datasets to test stationarity of series. For the identification of AR and MA components, we have plotted the autocorrelation function (ACF) and partial autocorrelation function (PACF) respectively. The purpose of ACF is to examine correlation between a time series and their lagged numerical observations, whereas PACF plot do the same but by adjusting for the presence of intermediate lags. Apart from these, there exists some numerical based approach for selection of ‘p’ and ‘q’. These approaches include use of AIC and BIC statistic. The minimal values of these statistic among different combinations of ARIMA model, will be considered best.

Further, to extract some meaningful insights from available datasets, we have calculated the decadal and annual growth rates in IMR by the following relation,

2 $$\:Decadal/\:annual\:\:growth\:rate\:\left(\%\right)=\left(\:\frac{IM{R}_{n}}{IM{R}_{0}}-1\:\right)\times\:100$$

where, $\:{IMR}_{n}$ and $\:{IMR}_{0}$, represent IMR at end (current) year and beginning (previous) years respectively. For calculating decadal change, one should ensure that differences between current and previous years is at ten years apart. Similarly, we have calculated annual change (%) in IMR from the year 2010 onwards by considering this difference at a year apart in expression (2).

### Assessment of model’s performance

For the evaluation model’s performance, root mean square error (RMSE), mean absolute error (MAE), and mean absolute percentage error (MAPE) have been calculated. These measures provide some conclusive evidences in order to assess the performance of employed model [28]. In particular, these error metrics are calculated as,

3 $$\:RMSE=\sqrt{\frac{1}{n}\sum\:_{i=1}^{n}{({y}_{i}-\widehat{{y}_{i}})}^{2}}\:;$$

4 $$\:MAE=\frac{1}{n}\sum\:_{i=1}^{n}\left|{y}_{i}-\widehat{{y}_{i}}\right|\:;$$

and,

5 $$MAPE = \frac{1}{n}\sum\nolimits_{i = 1}^n {\left| {\frac{{{y_i} - \widehat {{y_i}}}}{{{y_i}}}} \right|}$$

where, $\:{y}_{i}\:and\:\widehat{{y}_{i}}$; i = 1, …., n, are mentioned to denote actual and predicted values of IMR. The smaller values of these error metrics suggest the executed model to be best fitted.

## Result and discussion

**Fig. 2:**
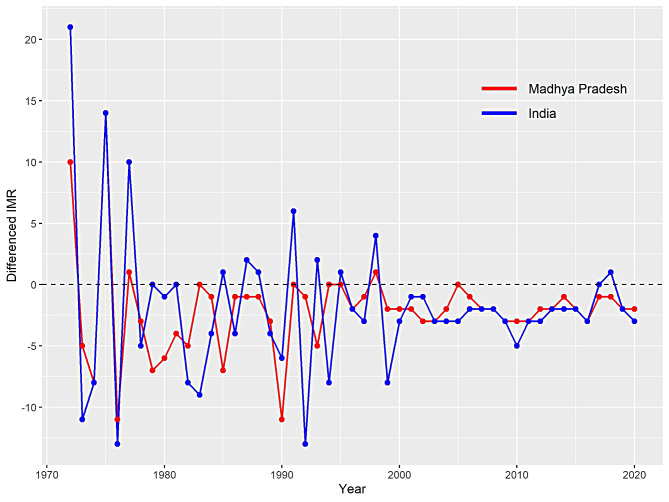
First differenced IMR series of India and Madhya Pradesh from 1971 to 2020

Time series models like ARIMA are widely used in various fields of research to analyse and forecast time-dependent data patterns as it provides a quick and effective approach for producing accurate forecasts. The existing literatures have shown that there are some important steps in execution of ARIMA model, for example, testing for stationarity, selecting ‘p’ and ‘q’, residuals’ diagnostics, and model validation, are among them. Thus, for the present study, the process of analysis of IMR datasets began with testing presence stationarity within it. In particular, the ADF and KPSS tests are used for this purpose [29, 30]. For ADF test, the null hypothesis is stated that IMR data of India and MP are non-stationary in nature. On the contrary, for KPSS test, the null hypothesis assumes the IMR datasets to be stationary. The results of these tests show that IMR series of India and MP are non-stationary in nature. However, after applying first order differencing on IMR data of India, we obtain stationarity with ADF test statistic equals to -4.913 (*p* < 0.01) and KPSS statistic equals to 0.07 (*p* > 0.05). From these findings, one can conclude that IMR data of India follows stationarity. Similarly, stationarity is obtained for IMR data of MP. The insights from these unit root tests are summarised in Table 1. Moreover, the resultant first order series of India and MP is depicted in Fig. 2. Now, one can observe that the differenced series is free from any kind of specific trend.

**Table 1:** Tests of stationarity on IMR data of India and Madhya Pradesh

Test	India				Madhya Pradesh			
	Before differencing		After differencing		Before differencing		After differencing	
	Value	*P*-value	Value	*P*-value	Value	*P*-value	Value	*P*-value
ADF	-2.011	0.569	-4.913	< 0.01	-5.857	< 0.01	-3.988	<0.01
KPSS	1.316	< 0.01	0.077	> 0.1	1.331	< 0.01	0.205	> 0.1

**Fig. 3:**
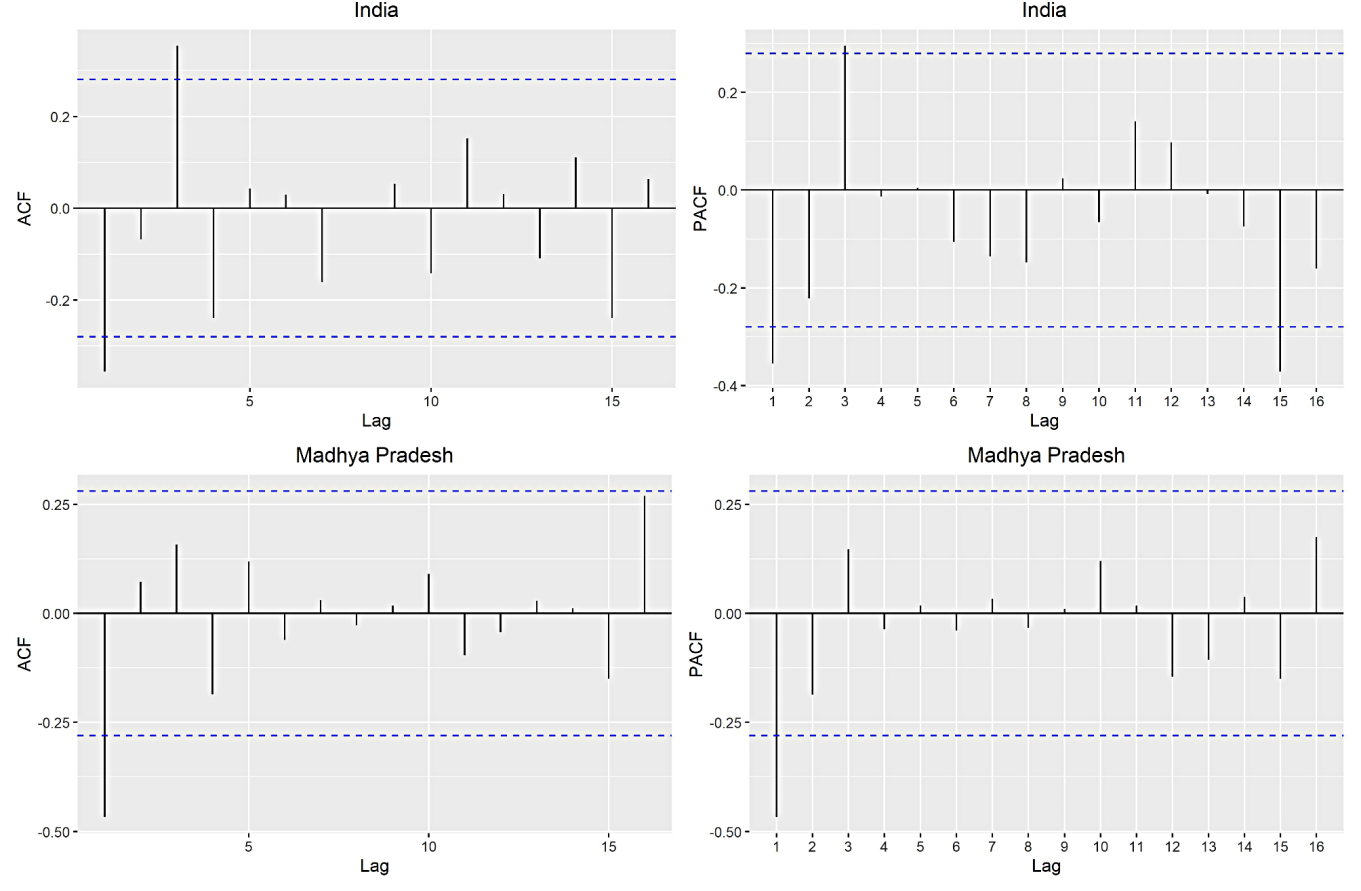
ACF and PACF plots on differenced series of India and Madhya Pradesh

**Fig. 4:**
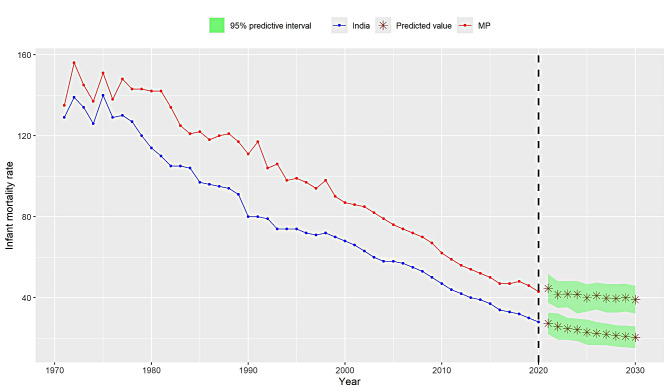
Projected IMR of India and Madhya Pradesh with 95% prediction interval

Since, stationarity is confirmed after first order differencing, therefore, ‘d’ is fixed to 1, in ARIMA process. Next, we seek to identify the order of AR and MA components. This objective can be achieved by ‘auto.arima( )’ command in R software. However, we plot ACF and PACF plots for this purpose (see Fig. 3). Both methods provide almost similar results. Apart from this, we use different combinations of ‘p’ and ‘q’ to select best performing ARIMA model, as represented in Table 2. Based on minimal values of AIC and BIC on differenced IMR datasets, we prefer to adopt ARIMA(3, 1, 0) model for data analysis. Furthermore, using ARIMA(3, 1, 0), we provide predicted values of IMR for India and MP from 1971 to 2020. The predicted IMR along with actual values (of same duration) are reported in Table 3. We obtain the residuals (errors) for these data points, and found performance of ARIMA(3, 1, 0) model to be satisfactory. Now, we consider it for future prediction of IMR. The findings revealed that estimated value of IMR for India is 27.36 (95% PI: 22.05–32.67) per thousand live births in the year 2021, which will reduced to 20.45 (95% PI: 15.13–25.89) per thousand live births by year 2030. Similarly, for MP, IMR will undergo a reduction from 44.59 (95% PI: 37.48–51.70) per thousand live births (in 2021) to 39.02 (95% PI: 32.14–45.90) per thousand live births by year 2030. These projected figures on IMR from 2021 to 2030 are presented in Table 4 and justified graphically (see Fig. 4) for readers’ better understanding.

**Table 2:** AIC and BIC values on first order differenced IMR datasets

Parameters	Model	India			Madhya Pradesh		
		Log likelihood	AIC	BIC	Log likelihood	AIC	BIC
*p* = 1, q = 1	ARIMA (1, 1, 1)	-142.31	290.63	296.3	-155.25	316.50	322.17
*p* = 1, q = 0	ARIMA (1, 1, 0)	-142.35	288.70	292.48	-155.34	314.68	318.46
*p* = 0, q = 1	ARIMA (0, 1, 1)	-142.36	288.72	292.5	-156.55	317.09	320.87
*p* = 2, q = 1	ARIMA (2, 1, 1)	-134.58	277.17	284.73	-149.16	306.32	313.88
*p* = 2, q = 2	ARIMA (2, 1, 2)	-134.39	278.79	288.24	-150.04	310.07	319.53
***p*** ** = 3,** ** q = 0**	**ARIMA (3**,** 1**,** 0)**	**-130.89**	**269.78**	**277.34**	**-147.07**	**302.15**	**309.71**
*p* = 3, q = 3	ARIMA (3, 1, 3)	-128.46	270.91	284.15	-144.57	303.13	316.37

**Table 3:** The obtained from between actual and predicted values of IMR from 1971 to 2020

Year	India			Madhya Pradesh		
	Actual	Predicted	Error	Actual	Predicted	Error
**1971**	129	128.87	0.13	135	134.86	0.14
**1972**	139	131.58	7.42	156	142.19	13.81
**1973**	134	136.47	-2.47	145	144.82	0.18
**1974**	126	133.63	-7.63	137	147.42	-10.42
**1975**	140	133.24	6.76	151	150.97	0.03
**1976**	129	131.64	-2.64	138	134.13	3.87
**1977**	130	129.55	0.45	148	144.28	3.72
**1978**	127	136.37	-9.37	143	147.38	-4.38
**1979**	120	120.76	-0.76	143	140.63	2.37
**1980**	114	121.60	-7.6	142	147.70	-5.7
**1981**	110	111.85	-1.85	142	139.39	2.61
**1982**	105	105.00	0	134	141.68	-7.68
**1983**	105	101.34	3.66	125	137.62	-12.62
**1984**	104	101.25	2.75	121	127.26	-6.26
**1985**	97	100.97	-3.97	122	115.27	6.73
**1986**	96	98.40	-2.4	118	114.56	3.44
**1987**	95	93.98	1.02	120	117.92	2.08
**1988**	94	90.44	3.56	121	118.30	2.7
**1989**	91	93.35	-2.35	117	118.58	-1.58
**1990**	80	90.82	-10.82	111	120.69	-9.69
**1991**	80	81.23	-1.23	117	113.55	3.45
**1992**	79	75.53	3.47	104	109.41	-5.41
**1993**	74	72.06	1.94	106	109.02	-3.02
**1994**	74	74.94	-0.94	98	104.61	-6.61
**1995**	74	72.20	1.8	99	94.71	4.29
**1996**	72	70.74	1.26	97	97.2	-0.2
**1997**	71	72.46	-1.46	94	93.35	0.65
**1998**	72	70.77	1.23	98	95.59	2.41
**1999**	70	70.23	-0.23	90	93.67	-3.67
**2000**	68	70.04	-2.04	87*	93.62	-6.62
**2001**	66	68.66	-2.66	86*	88.59	-2.59
**2002**	63	64.7	-1.7	85*	80.56	4.44
**2003**	60	61.94	-1.94	82*	83.33	-1.33
**2004**	58	58.71	-0.71	79*	82.65	-3.65
**2005**	58	55.82	2.18	76*	79.02	-3.02
**2006**	57	55.58	1.42	74*	74.76	-0.76
**2007**	55	55.93	-0.93	72*	72.23	-0.23
**2008**	53	55.23	-2.23	70*	70.55	-0.55
**2009**	50	52.36	-2.36	67*	69.17	-2.17
**2010**	47	48.94	-1.94	62*	66.71	-4.71
**2011**	44	45.71	-1.71	59*	62.45	-3.45
**2012**	42	42.06	-0.06	56*	57.13	-1.13
**2013**	40	39.82	0.18	54*	53.51	0.49
**2014**	39	38.05	0.95	52*	52.23	-0.23
**2015**	37	37.47	-0.47	50*	50.55	-0.55
**2016**	34	35.93	-1.93	47*	49.17	-2.17
**2017**	33	33.59	-0.59	47*	46.71	0.29
**2018**	32	31.24	0.76	48*	44.80	3.2
**2019**	30	30.05	-0.05	46*	45.58	0.42
**2020**	28	29.58	-1.58	43*	47.37	-4.37

*Chhattisgarh was formed from the state of Madhya Pradesh

Source of actual IMR: SRS bulletins, Registrar General and Census Commissioner, India

**Table 4:** ARIMA(3, 1, 0) based forecasts of IMR of India and Madhya Pradesh with 95% PI

Year	India			Madhya Pradesh		
	Forecast	95% prediction interval		Forecast	95% prediction interval	
		Lower value	Upper value		Lower value	Upper value
2021	27.36	22.05	32.67	44.59	37.48	51.70
2022	25.75	19.25	32.25	41.54	34.95	48.13
2023	24.67	19.21	30.09	41.78	35.29	48.27
2024	24.14	18.65	29.63	41.70	32.21	48.19
2025	22.97	16.77	29.17	39.90	33.22	46.58
2026	22.43	16.61	27.95	40.98	34.13	47.47
2027	21.94	16.49	27.29	39.79	32.74	46.84
2028	21.17	15.89	26.45	39.63	32.58	46.68
2029	20.88	15.55	26.21	40.02	33.13	46.91
2030	20.45	15.13	25.89	39.02	32.14	45.90

**Fig. 5:**
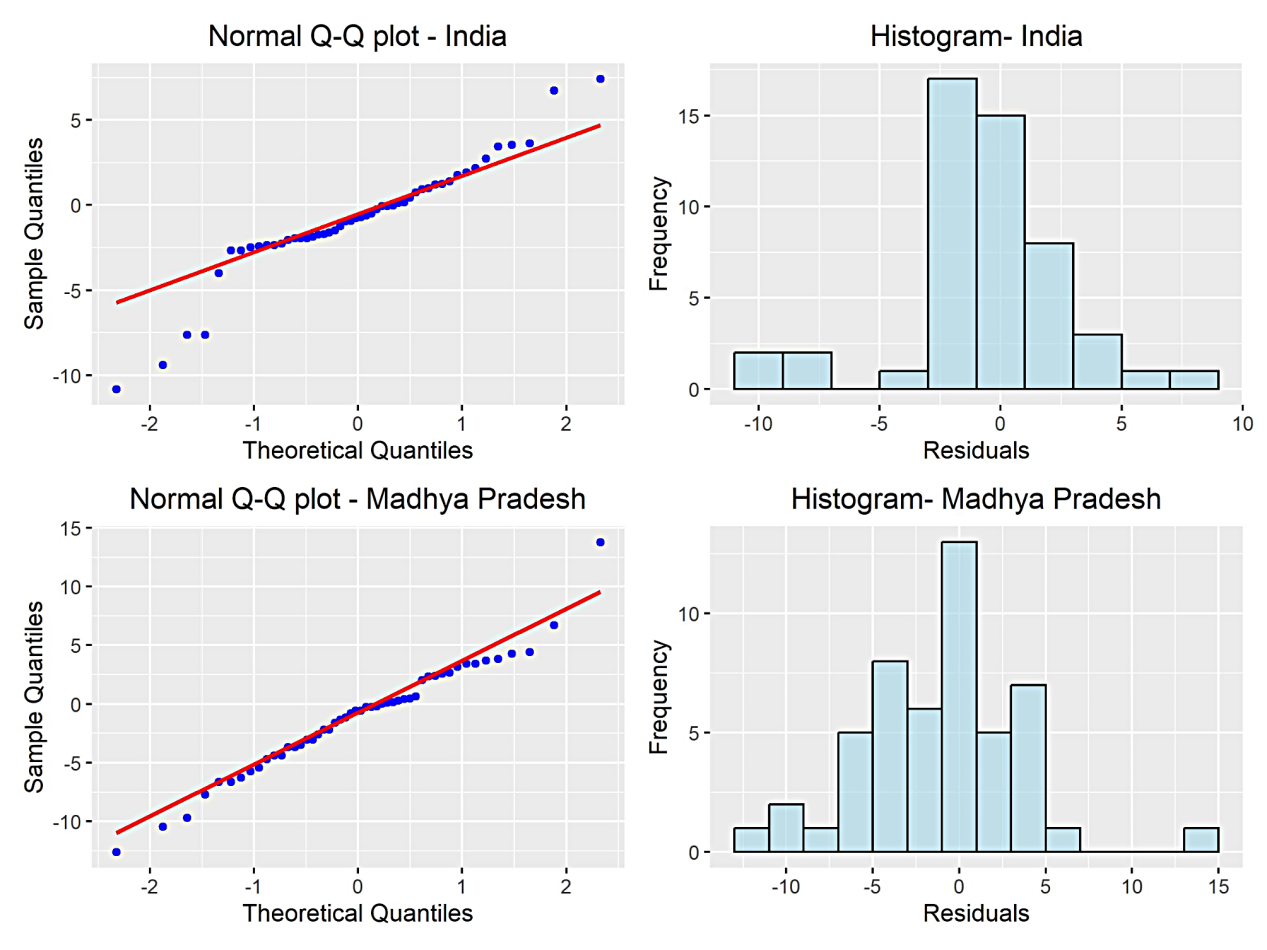
Residuals’ distribution for IMR series of India and Madhya Pradesh

Further, to assess the ability of ARIMA model, we perform residuals’ diagnostics on given IMR datasets. For this purpose, we provide quantile–quantile (Q-Q) plot and histogram in Fig. 5. It is clear from Fig. 5 that most of the observations fall near straight line and additionally, histogram seems to be bell shaped curve for IMR series of India and MP, supporting the fact that residuals are normally distributed. Besides, Ljung and Box test shows that p-value is greater than level of significance supporting the fact of no presence of autocorrelation in residuals [31]. The performance measures like MAE, RMSE, and MAPE possess smaller numerical values. The MAPE values for India and MP fall below 3% and 5% respectively (see Table 5). Therefore, we can conclude that implemented model is well fitted.

**Table 5:** Residuals’ diagnostics on IMR data of India and Madhya Pradesh

Metric	India	Madhya Pradesh
Root mean square error	3.40	4.72
Mean absolute error	2.39	3.52
Mean absolute percentage error	2.95	3.56
Ljung Box statistic	*p* > 0.05	*p* > 0.05

**Fig. 6:**
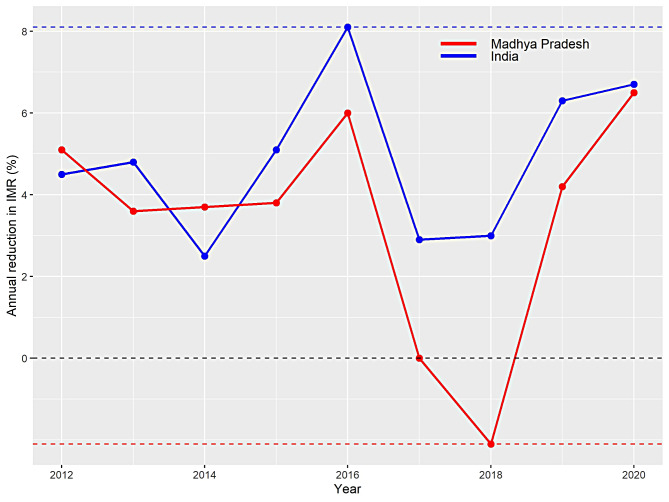
Annual reduction in IMR of India and Madhya Pradesh from year 2010 onwards

To have much clarity about IMR, we provide decadal reduction rate in Table 6, indicating the rate was 15% during 1990–2020, but now it has gone up to 40% during 2010–2020. Similarly, for MP, we observe that decadal reduction is moving steady and the highest reduction of 31% is observed recently in 2010–2020. Besides it, we provide change in annual growth rate from the year 2010 onwards. We observe that India experienced a highest annual reduction of 8.1% in IMR during 2015-16, while for MP this rate was 6% during the same period (see Fig. 6). However, a little warning sign was observed during 2017-18, when MP experienced growth of 2.1% in annual IMR, but this rate had started declining thereafter, and was observed a reduction of 6.5% during 2019-20. This reduction rate has been found highest in MP in the last ten years. On the other hand, India experienced a reduction of 6.7% for the same period. In relevance to the present findings, De et al. [32] showed the rapid reduction in under five mortalities as compared to neonatal mortality among Indian states. However, the study conducted by Pal et al. [33] reported that MP is behind by five years to the target set up in National health policy-2017. Low literacy rates amongst women, low median age at marriage, childbirth with one-fourth women marrying before attaining maturity, short birth intervals with two-thirds of all births within three years of the previous birth contribute to neonate deaths in MP. Vaccination dropouts, delayed breastfeeding, high rates of stunting, wasting and underweight infants, poor sanitation, high rate of childhood infections, poor maternal literacy rates, low maternal BMI, sub-par economic autonomy, shortfall in human resources and health facilities and poor access and utilization of health services are responsible for slow decline in infant mortality rates [19, 34–41]. For the present study, we found that despite an expected reduction in infant mortality, MP will remain above national average for IMR in coming years. To match the country’s pace, a revamp in maternal and child health services, education, nutrition and gender equality policies is the need of the hour [41, 42]. The state must refocus on quality care through Janani Shishu Swasthya Karyakram (JSSK), Janani Suraksha Yojana (JSY), Navjaat Shishu Suraksha Karyakram (NSSK), Home Based New Born Care (HBNC), Universal Immunisation, Integrated Management of Neonatal and Childhood Illnesses (IMNCI), Nutritional Rehabilitation Centres (NRCs), Rashtriya Bal Swasthya Karyakram (RBSK), Direct Benefit Transfers (DBT) under schemes like Pradhan Mantri Matru Vandana Yojna and Mukhya Mantri Ladli Behna Yojna (LBY), and educational reforms like Ladli Laxmi Yojana and Beti Bachao, Beti Padhao Yojana and others, to see gains in terms of newborn survival [19, 43–48].

**Table 6:** Decadal reduction (%) in IMR of India and Madhya Pradesh

Year	India (%)	Madhya Pradesh (%)
1980	-	-
1990	29.8	21.8
2000	15.0	21.6
2010	30.8	28.7
2020	40.4	30.6

## Conclusion

Future projection of IMR can prove to be an excellent means for a nation’s growth and setting up direction to health policies, especially pertaining to children’s health. Since various factors including preterm birth complications, low birth weight, infections during pregnancy, maternal health issues, lack of resources at local hospital, awareness, nutritional factors, etc., can affect IMR particularly in a developing country like India, therefore, addressing these issues at a right time can be helpful in managing the burden of excessive infant mortality and exploring the risk factors responsible for this rapid pace. But one of the challenges with demographic variables like IMR, is that forecast result cannot be predicted with 100% accuracy for a longer duration. So, it becomes a challenging task for researchers, especially with selection of a statistical model. However, in this regard, this study is an attempt to forecast IMR for India and MP for the next ten years using ARIMA model. The findings of the study compared the performance of MP to India in terms of the pace of reducing infant deaths by the year 2030. The figures on IMR are projected to be reduced from 27 (in 2021) to 20/1000 live births (in 2030) in India, and 45 (in 2021) to 39/1000 (in 2030) live births in MP. These declining figures are satisfactory for policy makers at national level, but MP is still miles away to achieve acceptable IMR as compared to the country’s IMR. We recommend the future researchers working towards infant deaths to give more attention towards the identification of risk factors responsible for higher IMR at national level and more especially, in MP. In addition to these, similar predictive models can be applied to different health datasets so that better action could be planned in due course.

## Limitations of the study

The study presents a projection of IMR in India and MP by the year 2030. These predictions can be helpful to veer the policy makers to create policies well-tailored to meet the needs of the nation and state. In addition, the study highlights the need of policy revisions and identification of various factors responsible for higher IMR in MP. The limitation of the study is that it only predicts future IMR but cannot pinpoint the risk factors of infant deaths and accordingly prevention strategies which could be more fruitful. The study is completely based on secondary time series data on IMR and does not discuss about the scenario of change in population dynamics of MP, after formation of Chhattisgarh state from it in 2000. However, for the reader’s understanding, the actual IMR data of MP after the said period is highlighted with asterisk (*) sign in Table 3. Also, prediction of IMR in longer run can be affected due to various factors like population fluctuation, policy change, literacy rates, and women’s autonomy etc. A complete availability of dataset on religion, caste, gender, rural-urban differentials, socio-economic status, comorbidities, parental history, etc., would have been beneficial, especially for paediatricians in terms of exploring underlying causes for infant deaths. This information could be helpful in providing a better picture of ongoing trend on child mortality and paradigm shift in policy implications.

## Data Availability

The present study is based on secondary data on IMR of India and MP, publicly available at Open Government Data (OGD) Platform, India (https://data.gov.in/catalog/infant-mortality-rate-india#web_catalog_tabs_block_10).

## Abbreviations

IMR: Infant Mortality Rate
ARIMA: Autoregressive Integrated Moving Average
MP: Madhya Pradesh
MDGs: Millennium Development Goals
SGDs: Sustainable Development Goals
AIC: Akaike’s Information criterion
BIC: Bayesian Information criterion
MAE: Mean Absolute Error
RMSE: Root Mean Square Error
MAPE: Mean Absolute Percentage Error
95% PI: 95% Prediction Interval
